# Editorial: Exploring ‘omic' biomarkers in animal production and reproduction

**DOI:** 10.3389/fvets.2025.1688780

**Published:** 2025-09-23

**Authors:** Pedro M. Aponte, Miguel A. Gutiérrez-Reinoso, Manuel García-Herreros

**Affiliations:** ^1^Colegio de Ciencias Biológicas y Ambientales (COCIBA), Universidad San Francisco de Quito (USFQ), Quito, Ecuador; ^2^Instituto de Investigaciones en Biomedicina “One-Health”, Universidad San Francisco de Quito (USFQ), Campus Cumbayá, Quito, Ecuador; ^3^Facultad de Ciencias Agropecuarias y Recursos Naturales, Carrera de Medicina Veterinaria, Universidad Técnica de Cotopaxi (UTC), Latacunga, Ecuador; ^4^Laboratorio de Biotecnología Animal, Departamento de Ciencia Animal, Facultad de Ciencias Veterinarias, Universidad de Concepción (UdeC), Chillán, Chile; ^5^Instituto Nacional de Investigação Agrária e Veterinária (INIAV), Santarém, Portugal; ^6^CIISA-AL4AnimalS, Faculty of Veterinary Medicine, University of Lisbon, Lisbon, Portugal

**Keywords:** genomics, transcriptomics, proteomics, metabolomics, animal production, animal reproduction

## Introduction

Animal reproduction and breeding are advancing at an unprecedented pace, closely aligned with technological innovation. While the field has historically relied on phenotypic selection to improve traits of interest, the emergence of high-throughput “omics” technologies (genomics, transcriptomics, proteomics, and metabolomics) is opening new possibilities for understanding the molecular basis of productivity, fertility, health, and welfare (1–5). Omics, especially when integrated with Assisted Reproductive Technologies (ARTs), enable the identification of precise biomarkers that can guide selection, diagnosis, and intervention strategies. The present Research Topic brings together recent advances aimed at uncovering and applying molecular indicators to enhance reproductive efficiency and overall performance in diverse animal production systems.

The diversity of species represented, from dairy goats and sheep to cattle, buffalo and avians (such as hens and geese), underscores the global relevance and adaptability of these tools. Collectively, the featured studies reflect an international effort to harness molecular insights for a more efficient, resilient, and sustainable livestock production.

## Genomics and transcriptomics

Key contributions demonstrate how genomic and transcriptomic analyses can uncover fundamental regulatory mechanisms, identify novel molecular markers, and inform breeding strategies to improve reproductive efficiency across species.

Shi et al. investigated the seasonal reproductive physiology of dairy goats by integrating transcriptomic and proteomic analyses of ovarian tissues collected during breeding and non-breeding seasons. Their work revealed marked reductions in gonadotropin levels and follicular size during the non-breeding period, underpinned by over 1,000 differentially expressed genes and more than 500 differentially expressed proteins. Common molecules, such as *TMEM205, TM7SF2, SLC35G1, GSTM1*, and *ABHD6*, were identified as potential mediators of suppressed follicular development via steroid hormone biosynthesis pathways.

Turning to poultry, Xiong et al. focused on the regulatory role of non-coding RNAs (ncRNAs) in ovarian atresia associated with broodiness in hens—a behavior detrimental to egg production. Using whole-transcriptome sequencing, they identified hundreds of differentially expressed MicroRNAs (miRNAs), Long Noncoding RNAs (lncRNAs), and circular RNAs (circRNAs), and constructed a competing endogenous RNAs (ceRNAs) network. Candidate genes such as *THBS1* and *MYLK*, which are regulated by specific miRNAs and circRNAs, were linked to pathways involved in ovarian function, including extracellular matrix (ECM)–receptor interactions, cytokine signaling, and hormone secretion.

From a structural genomics perspective, Zhao et al. examined copy number variations (CNVs) in Guizhou Black goats with divergent litter sizes. Through genome-wide selection signal analysis, they identified 180 CNVs and 49 candidate genes enriched in critical fertility pathways, such as Hippo signaling, steroid hormone biosynthesis, and retinol metabolism, highlighting the potential of CNVs as valuable genomic markers for selective breeding to enhance prolificacy in goats.

Finally, Xi et al. profiled miRNA expression across key developmental stages of sheep testes, from birth to maturity, identifying over 1,200 known and novel miRNAs. Differential expression analyses and pathway enrichment identified target genes such as *YAP1, ITGB1*, and *SOX9* in reproductive signaling pathways, including the FOXO, Hippo, Wnt, and MAPK pathways.

## Proteomics

Recent advances in proteomic technologies have enabled the identification of protein signatures that underpin reproductive physiology. These signatures offer valuable biomarkers for fertility assessment and improvement in livestock and avian species. Maulana et al. provided the first comprehensive proteomic characterization of seminal plasma and sperm in Toraya buffalo, revealing four key proteins—ADAM32 in seminal plasma and ZPBP, SPACA3, and CCDC136 in sperm—that are integral to motility, energy production, and acrosome formation. These findings, coupled with the enrichment of proteins in the tricarboxylic acid (TCA) cycle, highlight the metabolic underpinnings of sperm function in this indigenous Indonesian breed. Turning to avian reproduction, Yuan et al. explored lipid droplet-associated proteins (LDAPs) in the granulosa cells of goose follicles at hierarchical and pre-hierarchical stages, uncovering ACSL3 as a potential regulator of lipid metabolism via the fatty acid degradation pathway.

Proteomic biomarkers have also been employed to model and assess reproductive stress conditions. Liang et al. established an oxidative stress mouse model induced by hydrogen peroxide, profiling serum proteins alongside oxidative damage markers. This approach revealed shifts in antioxidant enzyme activities (CAT, SOD, and GSH-Px) and structural ovarian changes, providing a framework for studying protein-mediated stress responses that affect fertility. Finally, in Simmental bulls, Satrio et al. demonstrated that age significantly shapes the sperm proteome, with younger bulls expressing proteins linked to acrosome assembly (SPACA1) and spermatid development, middle-aged bulls showing markers of motility (PEBP4) and decapacitation (PEBP1), and older bulls exhibiting proteins related to capacitation-associated hyperactivity (Tubulin).

## Metabolomics

Metabolomics approaches are increasingly revealing the small-molecule signatures that reflect reproductive efficiency, stress resilience, and metabolic health in livestock, offering prospects for non-invasive monitoring and targeted interventions. In the context of bovine reproduction, Su et al. profiled serum metabolites at three key stages of superovulation: before FSH injection, before insemination, and before embryo collection. They identified consistent differences between high- and low-yield donors. Lipid-related metabolites, including specific phosphatidylcholines, phosphatidylethanolamines, triacylglycerols, phosphatidylinositols, and phosphatidylserines, emerged as candidate biomarkers linked to amino acid and fatty acid metabolism and ovarian steroidogenesis, providing a biochemical basis for donor selection in embryo transfer programs.

Zang et al. employed a metabolomics approach to investigate how cryopreservation disrupts mitochondrial energy metabolism in goat sperm. They documented structural mitochondrial damage, ATP depletion, and oxidative stress, alongside marked reductions in key energy- and antioxidant-related metabolites such as capric acid, creatine, and cholesterol sulfate. Functional validation showed that supplementing freezing extenders with capric acid significantly improved post-thaw motility, demonstrating a direct link between metabolite supplementation and fertility preservation.

At the herd-management scale, Magro et al. investigated metabolic profiling in dairy cows during the high-risk transition period by comparing invasive blood sampling with milk mid-infrared (MIR) spectroscopy. They found that MIR could moderately predict blood β-hydroxybutyrate and non-esterified fatty acids, which are critical indicators of negative energy balance, and accurately predict urea levels. Although MIR is not yet precise enough for individual diagnostics, its utility for herd-level screening and genetic evaluation underscores the role of metabolomics in large-scale, non-invasive metabolic health monitoring.

Taken together, the manuscripts in this Research Topic highlight how genomics, transcriptomics, proteomics, and metabolomics are converging to provide innovative insights into reproductive biology and animal sciences. Genomic and transcriptomic studies reveal regulatory mechanisms and potential molecular markers, while proteomics identifies protein networks that underpin fertility, and metabolomics captures physiological and metabolic signatures linked to reproductive efficiency and stress. By covering species ranging from goats and cattle to poultry and buffalo, this collective work demonstrates both the breadth of application and the translational potential of omics biomarkers across animal industries.

## Conclusion and perspectives

The “omics revolution,” which spans genomics, transcriptomics, proteomics, metabolomics, and now epigenomics, along with single-cell and spatial approaches, has transformed animal reproduction and production science by providing unprecedented insight into complex biological processes. The manuscripts in this Research Topic exemplify how these tools have already advanced the identification of molecular markers linked to fertility, prolificacy, stress resilience, and metabolic health across diverse species. Current applications are tangible: Single Nucleotide Polymorphisms (SNPs) and CNVs are guiding breeding programs; proteomic and metabolomic profiles are informing donor selection and cryopreservation strategies; and non-invasive metabolic screening is beginning to support herd-level management. These advances demonstrate that omics biomarkers are no longer confined to the discovery stage and are progressively entering applied contexts in assisted reproduction, nutritional monitoring, and fertility preservation.

Looking ahead, the challenge lies in moving beyond isolated datasets to integrated, hypothesis-driven, multi-omics research that bridges molecular discovery with functional validation. Progress will depend on strengthening species-specific functional databases, developing standardized protocols for reproducibility, and embracing computational advances, such as artificial intelligence and machine learning, to process complex datasets and enhance predictive modeling. The integration of “omics” with precision livestock farming and international collaborative frameworks, such as the Functional Annotation of Animal Genomes (FAANG) Consortium initiative, will accelerate genome-to-phenome understanding and the design of targeted interventions. By aligning these technological advances with the goals of sustainability, climate resilience, and animal welfare, the field is poised to deliver transformative impacts, shaping future livestock systems that are not only more productive and efficient but also more ethical and robust in the face of emerging global challenges.
